# Nasal Catarrh, with Especial Reference to the Correction of the Fetor of Ozaena

**Published:** 1898-01

**Authors:** Edward B. Jackson

**Affiliations:** Houston, Texas


### Nasal Catarrh, with Especial Reference to the Correction of the Fetor of Ozaena.

BY EDWARD B. JACKSON, M. D., HOUSTON, TEXAS.

There are so many stages of rhinitis, namely: acute, chronic, hypertrophic, atrophic, and, we may add, the necrotic, which furnishes *stinknase*—Ger.—that it will not here be endeavored to detail the varying anatomical characters. It is sufficient to be reminded of the salient marks along the burrowing track of this old enemy; and after alluding to the most important points concerning the different phases of the disease, we will undertake to speak more particularly of the extreme stage—ozæna.

Preparatory to the fullest comprehension of the pathological routes which are made in the rhinitic field, it may be stated that the journey is begun, in the simple, commonplace words, catching cold. i. e., coryza, sneezing, congestion of the nasal mucosa, turgescence of the erectile tissue of the nares, engorgement of the septum, which may be so extensive as to preclude the possibility of the respiratory function through the natural channel.

Some hours—usually not exceeding forty-eight—after these conditions are seen to prevail, a sero-mucoid leakage is established, the general tumefaction is relieved, febrile movement subsides, and the case is ready to merge into chronic rhinitis, unless it be immediately changed with intelligent treatment.

These attacks, we believe, are oftenest the result of transition from warm to cold—and vica versa—surroundings, though they are lamentably often the result of exanthematous febrile manifestations. Occasionally they come as the result of breathing pungent air laden with irritating particles, such as are furnished by certain drugs, metals, and the pollen of plants. The gouty detritus in the blood of a gormand may often be the initial factor in igniting the nostril into inflammatory action.

It will be readily seen that the disease, if permitted to progress, will shortly untone the blood vessels; will produce a hyperplastic condition of the erectile tissue; will chronically thicken the epithelial lining, blunting permanently its valued function, and will extend probably further into adjacent quarters, engendering deafness, cough, sore throat, and hoarseness. If caries of the turbinated bones and necrotic invasion of the soft superstructures should become established, as an intercurrent diseased process, ozæna, with its diabolical stench, is the result.

### TREATMENT.

At first, when the disease is in its inception—the acute attack—rest in bed and sedatives are essential. Three drops of Battley's solution—liq. opii. sedative—every hour until five or six doses, as the case may require, will be found to act excellently. This, if we rightly recollect, is the old German treatment of cold, and we can assure its virtue if the patient be sent to bed.

The pain and swelling may be easily and harmlessly relieved by a 2 per cent. cocaine spray, which benumbs the sensibility of the Schneiderian membrane, and paves the way nicely for the antiseptic and astringent douche. We use glyco-thymoline, because it is deodorizing and agreeable to the patient. It favors the rapid resolution of the phlogistic process, fosters cell growth, and thus aids markedly in the repair of the eroded mucosa. The bowels must be regularly emptied, any taints of constitution which may, after a careful inquisition, be discovered must of course be summarily corrected by specific medication. Tonic remedies are often required for cases suffering unconsciously of latent malaria. There may be co-existence of gout, rickets, syphilis, scrofula, scurvy or lead poisoning, and it is needless to emphasize the painstaking effort which may be required in determining the origin of the lesion. When the case has matured into nauseous ozæna much systematic cleansing—by means of a disinfecting douche which shall reach the posterior nares and carry away the putrefying retained excretions, is necessary; in fact, indispensible. We doubt the utility of sprays and ointments so frequently used, for the very good reason that the one is, in our experience, inefficient, and the other cumbersome and nasty. We have tried sprays of chloride and bicarbonate of sodium; of carbolic and boric acid, and of many complicated formulæ too numerous to mention, and all seemed to be impotent to render the parts sterile. We have tried iodol, iodoform, phenic acid, zinc and murcury, in ointment tampons, placed alternately in the nostrils; but, as before stated, this is a distasteful procedure to the patient, and will not cure the case if the disease be already established in the posterior nares. It will likewise not remove the decomposing secretions; and every one readily concurs that all gummy scabs and necrotic flakes must come away, and the membrane rendered aseptic before any progress can intelligently be expected. An antiseptic fluid must be made to flow through nares and post nares, dislodging excrementitious debris, sterilizing the submucous denuded structures, and deodorizing the channel, thus insuring the aid which nature requires in rebuilding lost tissue. The character of detergent wash best adapted to this purpose is furnished by Kress—glyco-thymoline. There is nothing to equal the simplicity with which it is applied—with the Bermingham nasal douche—and it is a superior antiseptic and deodorizer. The first and most pronounced result of a glyco-thymoline nasal bath is the instant restoration of easy respiration through the nose; whereas before, there was probably dyspnœa, even during the process of breathing through the mouth. This agreeable remedy may be applied with safety to young children of scrofulous and rachitic habit, who often suffer with ozæna. The cost of the little crystal apparatus is nugatory, and since its convenience and simplicity surpasses anything which has yet come to our knowledge, we believe it should occupy a place in every household, accompanying the respective toothbrush. We believe and teach that the nasal cavity requires no less hygienic attention than the buccal and aural.

The object sought is regular and complete sterilization of the membrane so that nature may oppose and ward off diseased action. With proper care of the constitution and correct local measures of hygiene, the percentage of nasal affections—now enormous—can be surprisingly reduced.

